# *Polygonum cuspidatum* Extract Exerts Antihyperlipidemic Effects by Regulation of PI3K/AKT/FOXO3 Signaling Pathway

**DOI:** 10.1155/2021/3830671

**Published:** 2021-12-09

**Authors:** Ting Tao, Qing Zhang, Zibo Liu, Ting Zhang, Lingyu Wang, Jia Liu, Tao He, Yunhui Chen, Jiayue Feng, Yang Chen

**Affiliations:** ^1^Chengdu University of Traditional Chinese Medicine, Chengdu 611137, China; ^2^Department of Cardiovascular Medicine, Sichuan Provincial People's Hospital, University of Electronic Science and Technology of China, Chengdu 610072, China

## Abstract

*Polygonum cuspidatum* (PC) has been reported to exert a potent antihyperlipidemic effect. However, its mechanisms of action and active ingredients remain elusive and require further research. In this study, we first conducted *in vivo* experiments to validate that *Polygonum cuspidatum* extract (PCE) could ameliorate the blood lipid level in hyperlipidemia model rats. Then, ultrahigh performance liquid chromatography coupled with Q-Exactive MS/MS (UPLC-QE-MS/MS) was applied to verify its 12 main active ingredients. The pharmacophore matching model was employed to predict the target point of the active ingredient, and 27 overlapping genes were identified via database and literature mining. String online database and Cytoscape software were utilized to construct a Protein-Protein Interaction (PPI) network, followed by function annotation analysis and pathway enrichment analysis. The results showed that the PI3K/AKT signaling pathway and its downstream FOXO3/ER*α* factors were significantly enriched. Furthermore, *in vitro* experiments were performed to determine the lipid content and oxidative stress (OS) indicators in OA-induced HepG2 cells, and immunofluorescence and western blotting analysis were carried out to analyze the effects of PCE on related proteins. Our experimental results show that the mechanism of antihyperlipidemic action of PCE is related to the activation of the PI3K/AKT signaling pathway and its downstream FOXO3/ER*α* factors, and polydatin and resveratrol are the main active ingredients in PCE that exert antihyperlipidemic effects.

## 1. Introduction

Hyperlipidemia is a disease of lipid metabolism featured by elevated total cholesterol (TC), total triglycerides (TG), and low-density lipoprotein cholesterol (LDL-C) and reduced high-density lipoprotein cholesterol (HDL-C) in the peripheral blood. In recent years, the incidence of hyperlipidemia continues to rise due to decreased physical activities and increased consumption of fast foods high in calorie and low in fiber [1]. And current studies reveal that hyperlipidemia is a major risk factor for cardiovascular diseases [2]. Therefore, proper treatment of hyperlipidemia is of great significance. Clinically, its mainstay treatments include fibrates, statins, niacin, resins, and other lipid-lowering medications [3]. However, they may associate with treatment resistance and intolerance and cause numerous adverse effects, such as gastrointestinal symptoms, myalgia, and respiratory infections [4]. Hence, it is necessary to research and develop more therapeutic options for hyperlipidemia.

The dry rhizome and root of *Polygonum cuspidatum* Sieb. et Zucc (PC) can dispel dampness, eliminate jaundice, clear heat, detoxification, dispel blood stasis, relieve pain, arrest cough, and resolve phlegm [5]. It contains a variety of chemical compounds and has been widely used as a Chinese medicinal material to treat diseases such as inflammation and hyperlipidemia [6]. Modern pharmacological studies demonstrate that polydatin, one of the active ingredients in *P. cuspidatum* extract (PCE), can effectively reduce serum TC, TG, and LDL-C levels in the high-fat and high-cholesterol rabbit model and inhibit the activity of acyl-coenzyme a-cholesterol acyltransferase (ACAT) in a dose-dependent manner [7]. Xie et al. have reported that polydatin can significantly decrease the blood lipid level of hyperlipidemic mice [8]. Moreover, Kim et al. have validated that the ethanol extract of PC may inhibit pancreatic lipase activity and adipogenesis by downregulating lipid accumulation [9]. Besides that, using metabolomics approaches, researchers reported the association between the variation of specific urinary metabolites and the hypolipidemic effects of PC in rats after a prolonged treatment with PCE [10]. The antihyperlipidemic effects of PCE have been well-documented in several studies. However, its mechanisms of action and active ingredients are not fully understood and still need to be further elucidated.

In this study, we first performed *in vivo* experiments to validate the pharmacological activity of PCE in improving hyperlipidemia, then the original data chip was downloaded from the Gene Expression Omnibus (GEO) database, and the differential genes were screened by comparing the gene expression profiles in normal human and hyperlipidemic sample tissues. Furthermore, the main chemical components of PCE were identified by high-performance liquid chromatography-mass spectrometry technology, and the target points of the components of PCE were obtained through database and literature mining. Finally, the mechanism of PCE in the treatment of hyperlipidemia was systematically investigated via bioinformatics analysis, network pharmacology, and *in vitro* experiments. Hopefully, the findings of this study would provide a scientific basis for elucidating the mechanisms of the antihyperlipidemic activity of PCE and its effective ingredients.

## 2. Materials and Methods

### 2.1. Materials

Positive drug fenofibrate capsules (Lipingzhi) were produced by RECIPHARM FONTAINE in France (production batch number: 26763); carboxymethylcellulose (CMC)-Na was purchased from Chengdu Sinopharm Reagent Company (Chengdu, China); TG, TC, LDL-C, HDL-C, catalase (CAT), glutathione peroxidase (GSH-Px), trace reduced glutathione (GSH) detection kit, and oil red O dye were provided by Nanjing Jiancheng Institute of Bioengineering (Nanjing, China); sodium pentobarbital was obtained from Beijing Chemical Reagent Company (Beijing, China); 4% paraformaldehyde was purchased from Chengdu Kelon Chemical Reagent Factory (Chengdu, China); hematoxylin dye was purchased from Beijing Bailingwei Technology (Beijing, China); fetal bovine serum (FBS), phosphate-buffered saline (PBS), penicillin-streptomycin, trypsin-EDTA acid, and Dulbecco modified eagle medium (DMEM) were purchased from GIBCO (Grand Island, New York, U.S.); Cell Counting Kit-8 (CCK-8) detection kit was provided by Beijing 4A Biotechnology Co., Ltd. (Beijing, China); oleic acid (OA) was purchased from Xi'an Kunchuang Technology Development Co., Ltd. (Xi'an, China); Total Superoxide Dismutase (SOD) activity and malondialdehyde (MDA) assay kits were purchased from Biyuntian Biotechnology (Shanghai, China); primary antibodies for PI3K, phosphorylation (p)-PI3K, AKT, p-AKT, FOXO3, and ER*α* were obtained from the ImmunoWay Biotechnology Co. (Suzhou, China); RIPA lysis buffer, BCA protein concentration determination kit, SDS-PAGE gel preparation kit, SDS-PAGE protein loading buffer, blocking protein TBS-T buffer system blocking solution, and TBS-T rinsing buffer were obtained from Wuhan Boster Biological Engineering Co., Ltd. (Wuhan, China); UItraSignal ultrasensitive ECL chemiluminescence substrate was provided by Beijing Sizhengbo Biotechnology Co., Ltd. (Beijing, China).

### 2.2. Preparation of Freeze-Dried Powder of PCE

PC was purchased from Chengdu Niots Chinese Medicine Decoction Pieces Co., Ltd., China, and was identified by Professor Wu Chunjie from the School of Pharmacy of Chengdu University of Traditional Chinese Medicine. The sample of PC was deposited in the Chinese Medicine Specimen Museum of the School of Pharmacy of Chengdu University of Traditional Chinese Medicine (No.: 2019091701#). First, the powder of PC (126 g) was refluxed with 8 times 70% ethanol (1 : 8, *w*/*v*) for 30 minutes, and the filtrate was concentrated before removing the ethanol with a rotary evaporator, and then, the powdered extracts were obtained by freeze-drying the concentrated samples with the LGJ-12B freeze dryer (China Shanghai GIPP Co., Ltd.) and stored in a refrigerator at 4°C for the following animal experiments.

### 2.3. In Vivo Experiment

#### 2.3.1. Establishment and Administration of Hyperlipidemic Model Rats

Sixty female Wistar rats weighing 200 ± 20 g (5-8 weeks) were selected and purchased from Chengdu Dashuo Biotechnology Co., Ltd. (Chengdu, Sichuan; production license number: SCXK (Sichuan) 2015-030). All animal experiments were approved by the Animal Ethics Committee of Chengdu University of Traditional Chinese Medicine, and the experimental procedures have strictly followed the animal experiment management regulations. The 60 female Wistar rats were randomized into six groups, each with ten rats, namely, the normal group (normal), the model group (model), the positive drug group (positive), the low-dose group of PCE (low dose), and the middle group of PCE (middle dose) and the high-dose group of PCE (high dose). The normal control group was fed basic feed (Chengdu *Dashuo* Biotechnology Co., Ltd., production batch number: 20180501), and the other groups were fed high-fat feed. In addition to the basic feed, the high-fat feed contained sucrose, lard, egg yolk powder, etc. All rats were fed with free access to water and rodent chow and conditioned in a breeding room at 25 ± 2°C with a relative humidity of 55% ± 10% under a dark/light cycle of 12 h. After successful modeling, except for the normal group, rats in the other groups continued to receive the high-fat diet for 4 weeks and were given corresponding drugs according to the prespecified interventions, once a day by gavage for 4 weeks. The specific dosage regimen is shown in Table 1. The weight of the rats was measured regularly once a week, and the daily food intake and survival status of the rats were recorded.

#### 2.3.2. Collect Samples

After 4 weeks of administration, all animals were fasted for 12 hours. The rats were anesthetized with 2% sodium pentobarbital (3 mL/kg); blood samples were taken from the abdominal aorta to the disposable negative pressure blood collection tube. The sample was left at room temperature for 30 minutes, followed by centrifugation at 2500 rpm for 15 minutes. Then, the upper serum was taken and stored in -20°C frozen condition for analysis. After blood collection, the liver was dissected, and the regular part of the left lobe of the liver was taken and cut into 1 cm × 1 cm × 0.5 cm cubes with a razor blade prior to fixation in 4% formaldehyde solution.

#### 2.3.3. Determination of High-Fat Indicators in Plasma

The frozen serum samples were thawed at 4°C and then rewarmed at room temperature. The levels of TC, TG, LDL-C, HDL-C, and oxidized low-density lipoprotein (ox-LDL) were determined with a microplate reader following the instructions of the kit.

#### 2.3.4. Liver Tissue Morphology Analysis

The well-fixed tissue specimens were routinely dehydrated, embedded in paraffin, cut into 4-6 *μ*m sections, and stained using Hematoxylin and Eosin (H&E) for morphological observation with an optical microscope. The BA200 Digital trinocular microscope camera system was used to collect images. Each slice was first observed in 40 times magnification, and then, 400-fold images were collected to analyze the specific liver lesions in rats.

### 2.4. Qualitative UPLC-QE-MS/MS Analysis

A certain amount of freeze-dried PCE powder was weighed, dissolved in 70% methanol, ultrasonically treated for 40 minutes, allowed to cool to room temperature, and then centrifuged at 5000 rpm for 5 minutes. A 1.0 mL supernatant was taken and filtered with 0.22 *μ*m microporous membrane, and the filtrate was further diluted by methanol to a concentration of 0.2 mg/mL to obtain a sample of PCE for subsequent sample injection analysis.

For qualitative analysis, a Thermo Scientific Q Exactive Orbitrap HRMS (Thermo Fisher Scientific, Massachusetts, USA) was connected to a Thermo Scientific Vanquish UPLC (Thermo Fisher Scientific, Massachusetts, USA). Chromatographic separation was achieved on a Thermo ScientificTM AccucoreTM C_18_ (3 × 100 mm, 2.6 *μ*m) in a thermostatically controlled column compartment (30°C) [11].

The aqueous and organic mobile phases used were 0.1% formic acid in water (A) and methyl alcohol (B), respectively. The gradient elution system settings were as follows: 0-2 minutes, 0-20% B; 2-15 minutes, 20-40% B; 15-30 minutes, 40-60% B; 30-45 minutes, 60-90% B; 45-50 minutes, 90-5% B. The flow rate was 0.3 mL/min, and the injection volume was 2 *μ*L PCE. All samples were analyzed in both positive and negative ion modes, and the full scan was operated in the range of *m*/*z* 100-1500. For scanning analysis, the optimized parameter settings were as follows: sheath gas flow rate -35 L/min, spraying voltage -3000 V, capillary temperature -320 V, auxiliary gas flow rate -10.00 L/min, maximum injection current -100 A, probe heater temperature -350°C, and S-lens radio frequency level -50.00%. The main effective components of PCE were analyzed using liquid-mass spectrometry technology.

### 2.5. Target Retrieval of Chemical Constituents of PCE

The chemical components identified by UPLC-QE-MS/MS were searched in the PubChem database, and their two-dimensional structures were downloaded and saved in SDF format. These components were uploaded to the SwissTargetPrediction website to predict the potential targets of the compound. In addition, TCMSP data was used to collect target information for the main components of PCE.

### 2.6. Hyperlipidemia Target Collection

The GEO database is a database of gene expression that allows researchers to openly obtain experimental results of various microarray chips and high-throughput sequencing. In the GEO database, “hyperlipidemia” was used as the subject of the search, the species was set as Homo sapiens, and the gene expression profile dataset of “GSE1010” was collected. This dataset evaluates RNA samples prepared from lymphoblasts or cell lines from 12 normal individuals and 12 FCHL (familial combined hyperlipidemia) patients. GEO2R was utilized to perform background correction and data normalization on the dataset online, and *P* < 0.05 and |Log2FC | >1 were set as the screening conditions for significantly different genes. Finally, differentially expressed genes (DEGs) between hyperlipidemia and normal liver tissue were obtained. The ggplot2 package in the R language software was employed to visualize the results.

### 2.7. Venn Diagram Analysis of Drug Targets and Disease Targets

Furthermore, the DEGs obtained by GEO chip analysis and the drug prediction target were crossed to obtain the intersection gene.

### 2.8. PPI Protein Interaction Analysis

UniProt online database was applied to correct the standard names of these overlapping targets, these targets were uploaded to the String database, and the species were set as human origin to obtain the interaction relationship of these targets. Then, these interaction data pairs were downloaded and imported into Cytoscape software to construct PPI diagrams before using the CytoHubba plug-in to analyze pivot genes in PPI. In the network diagram, nodes represent targets, and edges represent interactions between proteins.

### 2.9. Drug-Active Ingredient-Target-Disease Network Construction

R language was utilized to construct the drug-active ingredient-target-disease data pairs, which were imported into Cytoscape software to draw the drug-active ingredient-target-disease network diagram. In the network diagram, nodes represent drug components and targets, and edges represent the correspondence between nodes. In addition, the network parameters were analyzed, including degree, average shortest path length, betweenness centrality, and closeness centrality of the node. And the importance of the node in the network graph was also evaluated.

### 2.10. KEGG and GO Analysis

Functional annotation and enrichment analysis on target genes were performed using the clusterProfiler toolkit of R language software, and the KEGG and GO functional enrichment analyses of overlapping genes were completed. The species were set as human, and the enrichment result of *P* < 0.05 was deemed as statistically significant. In addition, related histograms and bubble charts were provided.

### 2.11. In Vitro Experiments

#### 2.11.1. Cell Culture and Processing

Human hepatocellular carcinoma cell line HepG2 was purchased from Beijing Bena Biological Company (Beijing, China) and cultured at 37°C in a humidified atmosphere of 5% CO_2_ and 95% air in a sterile DMEM with 10% FBS and supplemented with 100 U/mL penicillin and 100 U/mL streptomycin.

#### 2.11.2. Cell Viability Test

The CCK-8 was used to detect the effect of PCE on HepG2 cells. In short, cells were seeded into 96-well plates (1 × 10^4^/well) and cultured at 37°C for 12 hours. Then, the cells were treated with different doses of PCE (0, 5, 10, 20, 40, 60, 80, and 100 *μ*g/mL) and cultured in the medium at 37°C for 24 and 48 hours [12], and 10 *μ*L CCK-8 was added to each well and incubated for 1 hour. In addition, HepG2 cells induced by OA (0.6 mM) were used to establish a cell model of hyperlipidemia, and the toxicity of PCE to HepG2 cells in the presence of OA was assessed according to the previous method. At the end of the experiment, a microplate reader was used to measure the absorbance of each well at 450 nm and calculate the cell survival rate. Each concentration of PCE had 3 multiple holes.

#### 2.11.3. Oil Red O Staining Analysis

**Figure 1:**
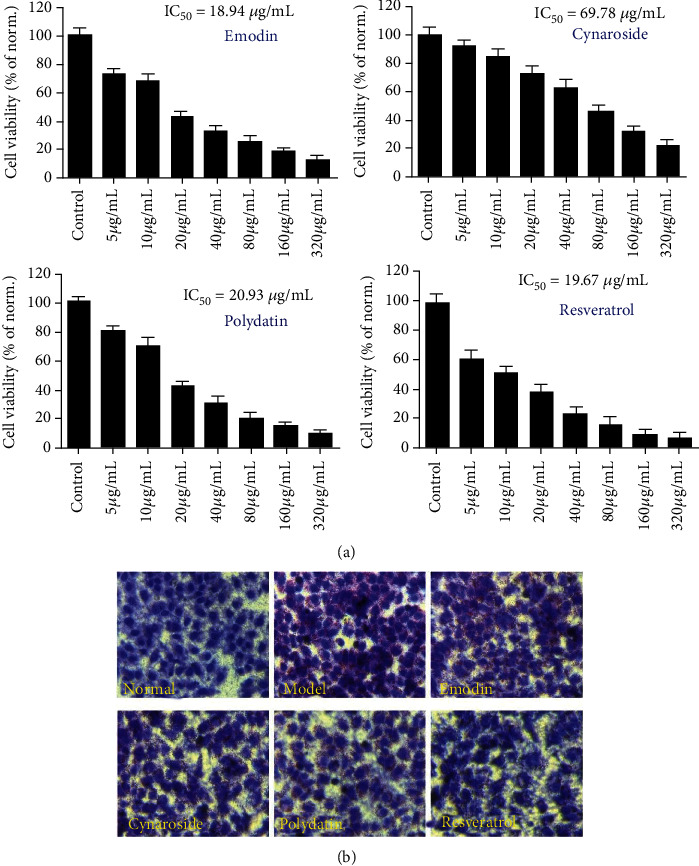
Lipid-lowering effects of emodin, cynaroside, polydatin, and resveratrol in OA-induced HepG2 cells: (a) effects of emodin, cynaroside, polydatin, and resveratrol on the proliferation of HepG2 cells; (b) oil red O staining results.

The cells in the logarithmic growth phase were seeded into a six-well plate and cultured for 12 hours, and then, OA (0.6 mM) and different doses of PCE (5, 10, and 20 *μ*g/mL) were added for treatment for 24 hours. In addition, according to our CCK-8 results and the IC_50_ value (Figure 1(a)), we selected the testing doses of all the compounds below the IC_50_ value. Therefore, emodin (10 *μ*g/mL), cynaroside (50 *μ*g/mL), polydatin (15 *μ*g/mL), and resveratrol (5 *μ*g/mL) were selected as the testing doses in our present study to observe the lipid-lowering effects of these monomers. At the end of the experiment, the cells were washed twice with PBS and then fixed with 4% paraformaldehyde for 15 minutes. After the fixation, the cell lipids and nuclei were stained with oil red O and hematoxylin, and the lipid accumulation in the cells was observed with a microscope. Moreover, photos were taken and recorded. In addition, two fluorescent dyes, Bodipy and Nile red, were used to stain lipids in cells, and confocal lasers were employed for observation and image capture.

#### 2.11.4. Determine the Content of SOD, MDA, CAT, GSH-Px, and GSH in Cells

Oxidative stress (OS) plays an important role in the occurrence and development of hyperlipidemia. Therefore, at the end of the experiment, the cell pellets were collected to measure the levels of SOD, MDA, CAT, GSH-Px, and GSH in the cells under the guidance of the commercial kit instructions.

#### 2.11.5. Detection of Reactive Oxygen Species (ROS) Accumulation in HepG2 Cells

Studies have shown that excessive ROS can cause DNA damage, enzyme inactivation, and lipid peroxidation, leading to inflammation, cardiovascular disease, and arteriosclerosis [13]. Therefore, DHE probe was used to detect intracellular ROS levels. DHE can freely penetrate the living cell membrane and enter the cell. Once it is oxidized by the ROS in the cell to form ethidium oxide, it will be incorporated into the chromosomal DNA of the cell and emit red fluorescence. The cells were intervened as described above, the supernatant was removed at the end of the experiment, and the cells were incubated with DHE (10 *μ*M) in a dark environment at 37°C for 20 minutes and then washed three times with PBS. The level of reactive oxygen species was analyzed by measuring the fluorescence intensity in the cell with flow cytometry.

#### 2.11.6. TG Determination

At the end of the experiment, after washing with PBS 1 or 2 times, the cells were collected and centrifuged at 1000 rpm/min for 10 minutes; then, the supernatant was discarded to collect the cell pellet. Then, the cells were lysed in RIPA lysis buffer and centrifuged, and the supernatant was collected. The concentration of TG in the cells was measured according to the instructions of the TG kit manufacturer.

#### 2.11.7. Immunofluorescence

At the end of the experiment, the cells were washed 3 times with precooled PBS, fixed with paraformaldehyde at -20°C for 20 minutes, washed 3 times with PBS buffer, and then incubated with 0.31% Triton for 30 minutes. After rinsing with PBS three times, 10% serum was added to block 1 h. Following serum aspiration, the sample was rinsed with PBS 3 times and incubated overnight with primary antibody dropwise p-AKT, AKT, FOXO3, and ER*α* (1 : 1000) at 4°C. Then, the primary antibody was discarded, the sample was rinsed with PBS 3 times and incubated with the diluted fluorescent secondary antibody (1 : 5000) dropwise for 1-2 h at room temperature. The secondary antibody was discarded, and the cells were rinsed with PBS 3 times and then imaged after adding the DAPI mounting tablets containing an anti-immunofluorescence attenuating agent and under a laser confocal microscope. The secondary antibody was discarded and washed with PBS for 3 times. Then, DAPI sealing agent containing an anti-immunofluorescence attenuating agent was added to the blocking agent and observed under a laser confocal microscope.

#### 2.11.8. Western Blot Analysis

At the end of the experiment, the total protein of HepG2 cells was extracted for WB analysis. In brief, 60 *μ*L of RIPA lysis buffer (add broad-spectrum protease inhibitor and broad-spectrum phosphatase inhibitor in a ratio of 100 : 1) was added to each small dish for 30 minutes, and then, the cleavage protein was collected and centrifuged under 12,000 rpm for 10 min at 4°C. The resulting precipitate was discarded, and the supernatant was saved. Then, the BCA protein concentration determination kit was utilized to determine the total protein concentration. Finally, protein sample was mixed 4 : 1 with loading buffer 5x, denatured by heating 4 minutes in a boiling water bath, cooled at room temperature, and stored at -20°C for later use. According to SDS-PAGE gel kit requirements, 10% separation and 5% concentration gels were prepared, and the separated target proteins were transferred to a polyvinylidene fluoride membrane (PVDF). After the transfer, the PVDF membrane was immersed in 5% fetal bovine serum solution and shaken gently on a shaker for more than 1 hour. The blocked PVDF membrane was washed with TBST solution 3 times (10 minutes each time) and incubated overnight with the corresponding primary antibodies PI3K, P-PI3K, AKT, p-AKT, and ER*α* (1 : 1000) dilution 5 mL at 4°C. Then, the PVDF membrane was washed 3 times with TBST solution (10 minutes each time) and incubated with secondary antibody (1 : 5000) for 1 hour at room temperature. After secondary antibody incubation, the membrane was washed 3 times with TBST (10 minutes each time). The protein bands were visualized using ECL reagent and quantitated using the ImageJ software.

### 2.12. Statistical Analysis

The data were expressed as mean ± standard deviation (SD), all statistical comparisons were evaluated by one-way analysis of variance (ANOVA), and significant differences between means were measured by Duncan's range test. *P* < 0.01 was deemed statistically significant.

## 3. Results

### 3.1. In Vivo Experiment

#### 3.1.1. Effect of PCE on the Morphology of Liver in Wistar Rats

**Figure 2:**
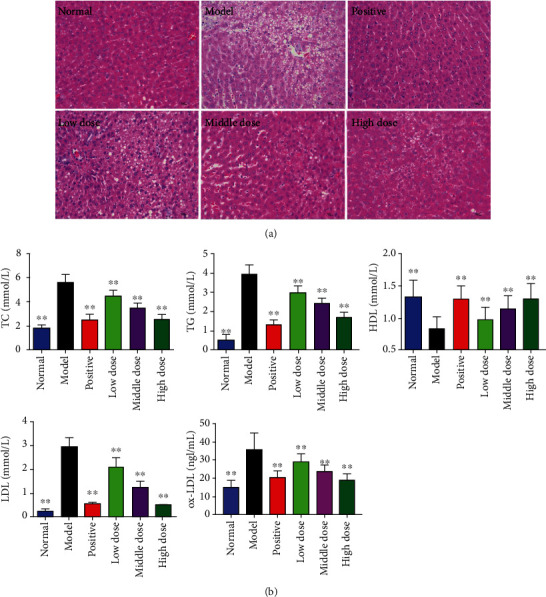
Effects of PCE on hyperlipidemia model rats: (a) HE staining results of liver tissue; (b) serum TC, TG, HDL-C, LDL-C, and ox-LDL levels in hyperlipidemia rats. Data were expressed as mean ± SD (*n* = 10), *P* < 0.01, vs. model.

As shown in Figure 2(a), compared with the normal group, the rats in the model group presented liver necrosis, interstitial edema, hepatocyte vacuolation, and serious accumulation of lipid droplets. The treatment of fenofibrate reduced the pathological changes of liver tissue; in particular, no obvious lipid droplets were observed in liver cells. Interestingly, similar to the effect of fenofibrate, PCE (90, 180, and 360 mg/kg) also improved the accumulation of lipid droplets in the liver cells of rats fed a high-fat diet.

#### 3.1.2. PCE Can Regulate Blood Lipid Levels in Hyperlipidemia Rats

As shown in Figure 2(b), compared with the normal rats, the serum levels of TC, TG, ox-LDL, and LDL-C in hyperlipidemia rats increased, and the level of HDL-C decreased (*P* < 0.01). Compared with the model group (hyperlipidemia rats treated with equal volume of CMC-Na), the positive drug group (*P* < 0.01) and the PCE high-, medium-, and low-dose groups (*P* < 0.01) significantly reduced the levels of serum TC, TG, ox-LDL, and LDL-C and increased the serum HDL-C level. The effects of different dose groups of PCE on improving blood lipids in the serum of rats gradually increased with the increase of dose.

### 3.2. Results of the Constituent Analysis of PCE by UPLC-QE-MS/MS

**Figure 3:**
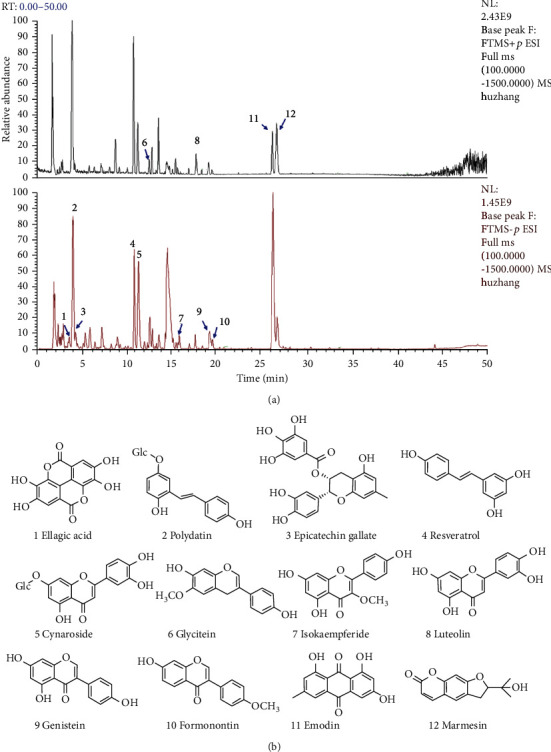
Result of the HPLC-Q Exactive-MS analysis of the PCE: (a) MS-BPI spectrogram (positive and negative mode); (b) a two-dimensional diagram of 12 compounds.

The main components in PCE were qualitatively analyzed using UPLC-QE-MS/MS. The analysis results are shown in Figure 3 and Table 2, including the positive and negative ion flow diagrams of UPLC-QE-MS/MS and the structural formulas of the main chemical components in PCE.

Within 50 minutes, the UPLC-mass spectrometry system detected more than 12 major component peaks from PCE. According to reported literature data, 12 major compounds were finally identified and inferred based on their mass spectrometry behavior and fragment ion characteristics. Finally, by comparing these components with standard reference compounds, the 12 main compounds were identified as ellagic acid (1), polydatin (2), epicatechin gallate (3), resveratrol (4), cynaroside (5), glycitein (6), isokaempferide (7), luteolin (8), genistein (9), formonontin (10), emodin (11), and marmesin (12).

### 3.3. The Target Prediction of PCE Improves Hyperlipidemia

**Figure 4:**
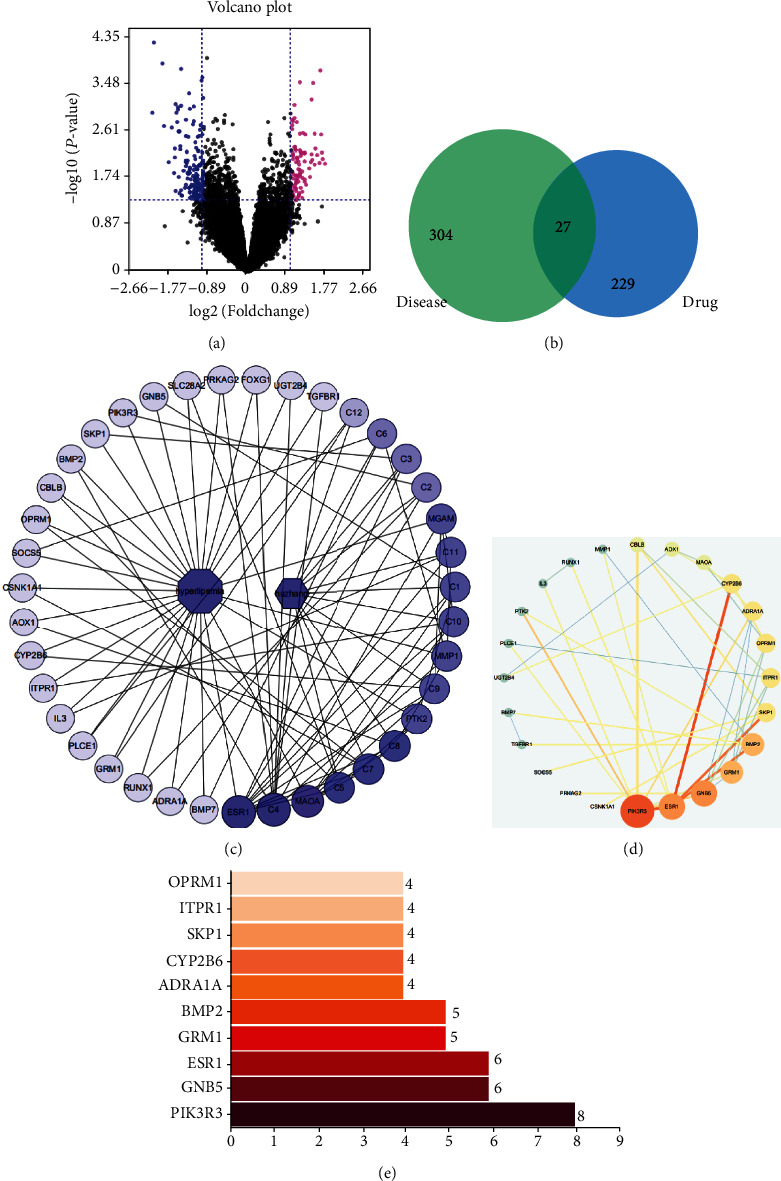
Differentially expressed genes (DEGs) identified: (a) volcano plot of the DEGs; (b) overlapped genes between DEGs and predicted targets of PCE; (c) compound-target network; (d) hub gene network; (e) the result parameters of the hub gene network.

The gene expression profile dataset “GSE1010” downloaded from the GEO database was analyzed and processed, and a volcano map of gene expression was obtained (Figure 4(a)). Finally, 331 differential genes (DEGs) were obtained in RNA samples prepared from lymphoblasts or cell lines of 12 normal persons and 12 FCHL (familial combined hyperlipidemia) patients, 114 of which were upregulated and 217 were downregulated genes. Comparing these differential genes with the predicted targets of PCE, a total of 27 overlapping genes were obtained (Figure 4(b)).

### 3.4. The PPI of PCE Improves Hyperlipidemia

String online database and Cytoscape software were used to construct a PPI network of overlapping genes. The network presented 24 nodes with 50 interaction edges. Through the analysis of the hub genes in the network, it was found that targets such as PIK3R3, GNB5, and ESR1(ER*α*) have higher MCC values, suggesting that these genes were important targets for improving hyperlipidemia in PCE (Figures 4(d) and 4(e)).

### 3.5. PCE Component-Target Network Diagram

As shown in Figure 4(c), the network diagram presented 39 nodes (12 compounds and 27 protein targets) with 180 edges, indicating the complexity of PCE in treating hyperlipidemia. Further in-depth analysis of the network graph revealed that a single compound could act on multiple targets, suggesting that the antihyperlipidemic effect of PCE was achieved by the interactions between multiple components and multiple targets. In addition, the analysis of the topological parameters in the network demonstrated that C4, C5, C7, C8, C1, C9, C10, C11, and other compounds occupied the core role in the network, indicating that these compounds were the main active components of PCE intervention in hyperlipidemia. Similarly, ESR1(ER*α*), MAOA, MGAM, PTK2, MMP1, GNB5, PIK3R3, and other targets had higher degree values, suggesting that these genes might be the core targets of PCE intervention in hyperlipidemia (Table 3).

### 3.6. GO Functional Enrichment Analysis and KEGG Signal Pathway Enrichment Analysis

**Figure 5:**
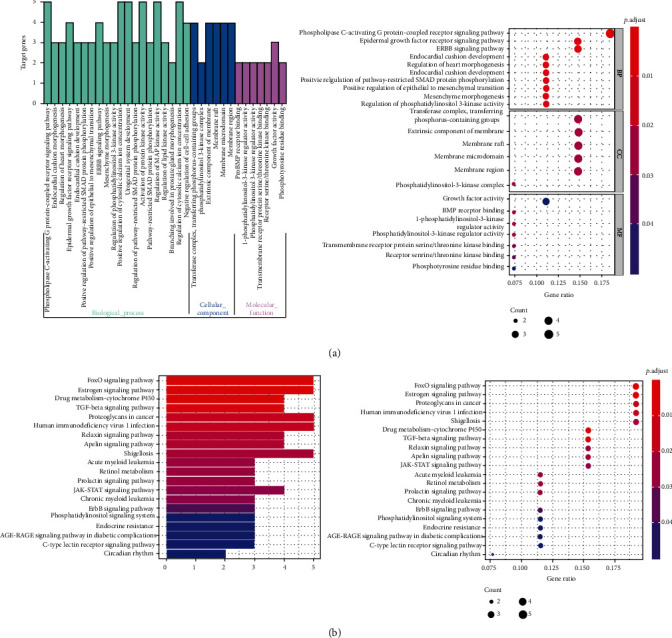
(a) GO functional enrichment analysis; (b) KEGG signal pathway enrichment analysis.

The GO function enrichment analysis of overlapping genes was performed. As presented in Figure 5, the top six biological processes (BP) significantly enriched by those overlapping genes were phospholipase C-activating G protein-coupled receptor signaling pathway, epidermal growth factor receptor signaling pathway, ERBB signaling pathway, positive regulation of pathway-restricted SMAD protein phosphorylation, positive regulation of epithelial to mesenchymal transition, and regulation of phosphatidylinositol 3-kinase activity. The top six significantly enriched cellular components (CC) included transferase complexes, transferring phosphorus-containing groups, membrane raft, membrane microdomain, membrane region, and phosphatidylinositol 3-kinase complex. The top six enriched molecular functions (MF) were growth factor activity, 1-phosphatidylinositol-3-kinase regulator activity, phosphatidylinositol 3-kinase regulator activity, transmembrane receptor protein serine/threonine kinase binding, receptor serine/threonine kinase binding, and phosphotyrosine residue binding.

KEGG signaling pathway enrichment analysis demonstrated that the main enriched signaling pathways were FOXO signaling pathway, estrogen signaling pathway, and drug metabolism-cytochrome P450. Among them, FOXO and estrogen signaling pathways were most closely related to hyperlipidemia (Figure 5).

### 3.7. In Vitro Experiments

#### 3.7.1. PCE Reduces OA-Induced Adipogenesis in HepG2 Cells

**Figure 6:**
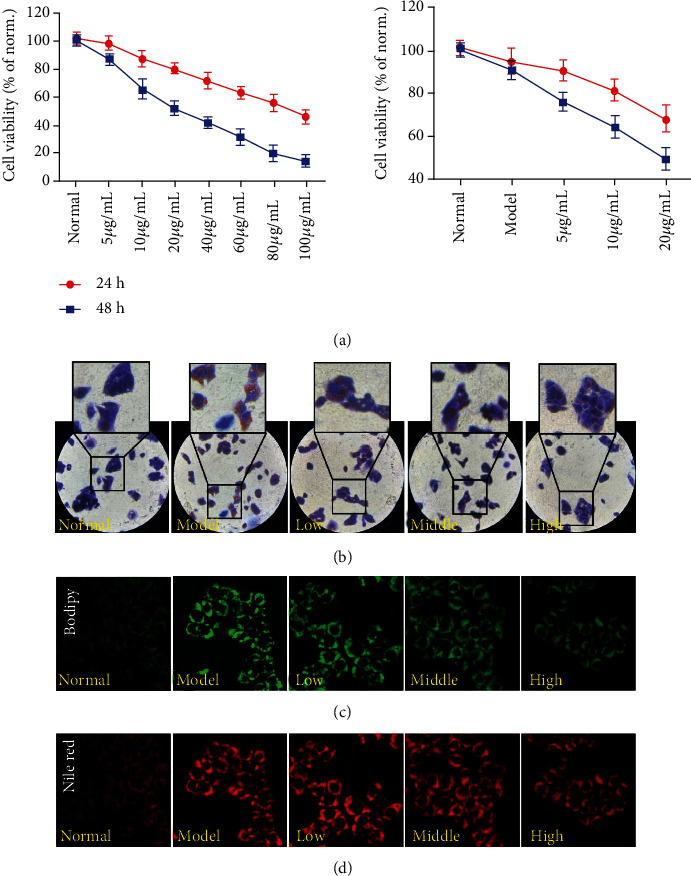
Lipid-lowering effects of PCE in OA-induced HepG2 cells: (a) effects of PCE on the proliferation of HepG2 cells; (b) oil red O staining assay; (c) Bodipy staining assay; (d) Nile red staining assay.

According to the experimental results of CCK-8, 5, 10, and 20 *μ*g/mL were selected as the safe dose of PCE for subsequent experiments. Meanwhile, the administration records also demonstrated no significant cytotoxicity to OA-induced HepG2 (Figure 6(a)). The effect of PCE and its active compounds on OA-induced adipogenesis was measured in HepG2 cells. As shown in Figure 6(b), oil red O (ORO) staining showed that HepG2 cells in the control group grew almost ovoid with clear edges, and obvious red lipid droplets gathering around the nucleus were evident in the cells of the OA-treated model group, indicating that the hyperlipidemia cell model was successfully constructed. The content of red lipid droplets in HepG2 cells showed a decreasing trend with the increase of PCE dose, and the lipid droplets became smaller and more intensely stained. As shown in Figure 6(c), the normal group without OA treatment had only weak green fluorescence, and the cells emitted strong green fluorescence after OA induction, indicating that the lipid content in the cells was significantly increased. While the fluorescence intensity in the cells decreased after PCE treatment, and as the PCE concentration increased, the green fluorescence intensity decreased more pronounced, indicating that OA could increase the content of intracellular lipid, and PCE could inhibit the lipid production induced by OA in a dose-dependent manner. In Figure 6(d), the lipids in HepG2 cells were stained with Nile red to emit red fluorescence. Compared with the normal group without OA induction, the model group showed stronger fluorescence intensity, and the fluorescence intensity gradually weakened with the increase of PCE dose. In addition, we also examined the therapeutic effects of some characteristic components of PCE on hyperlipidemia model cells, including emodin, cynaroside, polydatin, and resveratrol. In Figure 1(b), there were obvious red lipid droplets in HepG2 cells induced by OA. All four monomer treatments could reduce lipid production in HepG2 cells induced by OA.

All the above results suggested that PCE could significantly reduce the adipogenesis of HepG2 cells induced by OA and might have a certain preventive effect on hyperlipidemia. Among the compounds, resveratrol and polydatin have the strongest lipid-lowering effects, suggesting that resveratrol and polydatin may be the main active ingredients for PCE to lower blood lipids. These experimental results confirmed the predicted results of network pharmacology.

#### 3.7.2. PCE Reduces OA-Induced ROS Production in HepG2 Cells

**Figure 7:**
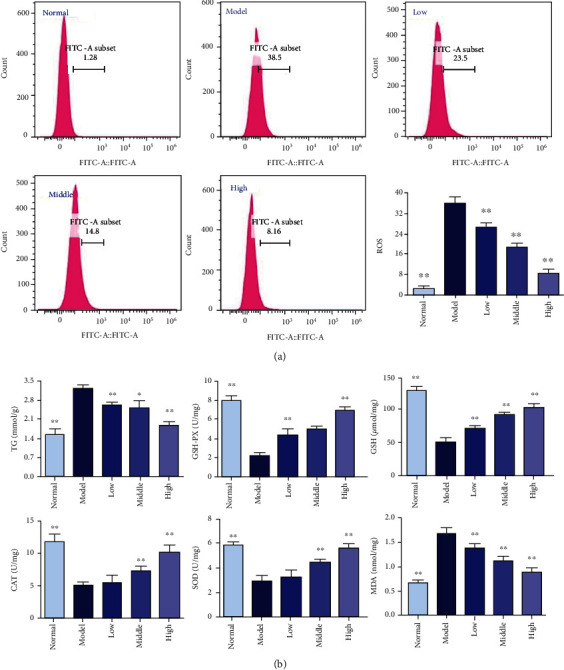
Effects of PCE on oxidative stress in OA-induced HepG2 cells. (a) The effect of PCE on the ROS level of OA-induced HepG2 cells. Values are expressed as mean ± SD (*n* = 3). (b) The effects of PCE on contents of TG, GSH-Px, GSH, CAT, SOD, and MDA in OA-induced HepG2 cells stimulated by OA. Values are expressed as mean ± SD (*n* = 3), ^∗^*P* < 0.01, compared with the control group.

Further, the fluorescent probe DHE was used to investigate whether PCE could inhibit ROS generation under OA stimulation and the OS caused by ROS. As shown by Figure 7(a), when the cells were treated with 0.6 mM OA, the ROS produced in the cells increased sharply compared with cells in the normal group (*P* < 0.01). However, compared with the model group, PCE (5, 10, and 20 *μ*g/mL) treatment significantly reduced the accumulation of ROS in HepG2 cells induced by OA (*P* < 0.01), and the accumulation of ROS gradually decreased with the increase of PCE dose.

#### 3.7.3. PCE Affects the Content of TG, GSH-Px, GSH, CAT, SOD, and MDA in HepG2 Cells Induced by OA

TG is a commonly used indicator for evaluating cell fat levels. As shown in Figure 7(b), the TG levels in the OA-induced model group and PCE (5, 10, and 20 *μ*g/ml) dose group were significantly higher than those in the normal group, suggesting that hyperlipidemic cells were successfully constructed. In addition, the TG level gradually decreased with the increase of the dose of PCE, indicating that PCE could reduce the production of TG.

Such biomarkers as intracellular GSH-Px, GSH, CAT, SOD, and MDA are commonly used to assess the level of OS in cells or tissues [14]. Therefore, to verify whether PCE has antioxidant properties that prevent OA-induced HepG2 cells from hyperlipidemia, the effects of PCE on MDA and GSH production and ROS scavenging enzyme (GSH-Px, CAT, and SOD) activities in OA-induced HepG2 cell influences were investigated. As shown in Figure 7(b), compared with the normal group of cells, the MDA content in HepG2 cells treated with 0.6 mM OA for 24 h increased significantly (*P* < 0.01). However, this growth trend was significantly reduced after 24 hours of PCE (5, 10, and 20 *μ*g/mL) intervention (*P* < 0.01) upon comparison with the model group, and the MDA content gradually decreased with the increase of the PCE dose. On the other hand, the total activities of GSH-Px, CAT, and SOD and the content of GSH in HepG2 cells induced by OA decreased drastically, and the PCE significantly increased the GSH-Px, GSH, CAT, and SOD in a dose-dependent manner. The number of antioxidant enzymes was positively correlated. The GSH-Px, CAT, and SOD levels in cells treated with high-dose PCE were basically the same as those of normal cells.

#### 3.7.4. PCE Regulates the Expression of p-AKT, AKT, and ER*α* in HepG2 Cells Induced by OA and Promotes the Transfer of FOXO3 to the Cytoplasm

The results of early network pharmacology suggested that the PI3K/AKT pathway and its downstream FOXO3 and ER*α* proteins were related to the improvement of hyperlipidemia by PCE. To further investigate whether the improvement effect of PCE on hyperlipidemia was associated with the regulation of the PI3K/AKT pathway, FOXO3 and ER*α* expression, western blotting, and immunofluorescence were performed to analyze the effect of PCE on p-AKT, AKT, ER*α* protein expression, and FOXO3 transfer.

**Figure 8:**
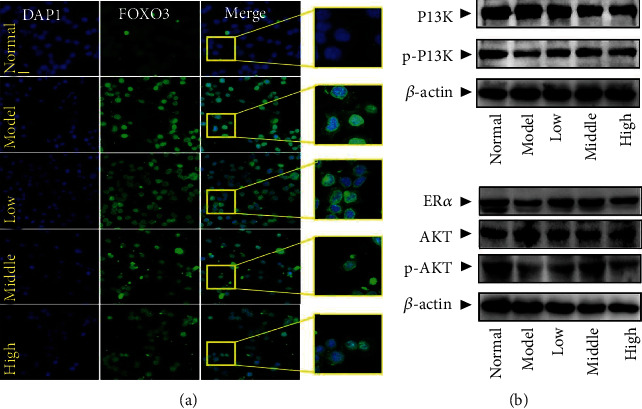
Effects of PCE on the PI3K-AKT signaling pathway and its downstream factors FOXO3 and ER*α* in OA-induced HepG2 cells. (a) The effect of PCE on the FOXO3 in HepG2. Under a laser confocal microscope (200x), the fluorescence intensity was detected with DAPI (blue) and FOXO3 antibody (green). (b) The effect of PCE on the expression of PI3K and p-PI3K, ER*α*, AKT, and p-AKT in HepG2. Western blot analysis results.

Modern research has revealed that FOXO3 is an important downstream gene of the PI3K-AKT pathway and plays vital roles in biological processes, such as OS and lipid synthesis. Activated AKT entering the nucleus can activate and phosphorylate FOXO3, reduce its DNA binding, and promote its transfer from the nucleus to the cytoplasm, thereby participating in biological activities such as cell OS, proliferation, and apoptosis. As shown in Figure 8, the fluorescence intensity of FOXO3 in the nucleus of the model group was significantly higher than that of the normal group after OA induction, indicating that the OA induction inhibited the transfer of FOXO3 to the cytoplasm, and the accumulation of FOXO3 in the nucleus increased. While after administration of PCE, the fluorescence intensity in the nucleus decreased in a dose-dependent manner, and the relative content of FOXO3 in the cytoplasm increased, indicating that PCE could promote the phosphorylation of AKT and induce the gradual transfer of FOXO3 to the cytoplasm.

**Figure 9:**
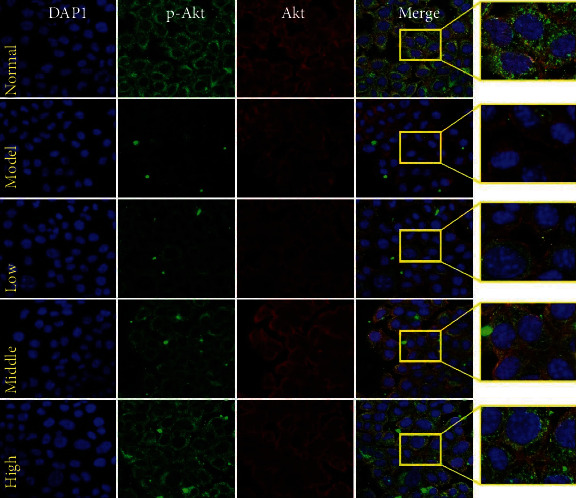
The effect of PCE on the expression of p-AKT and AKT in HepG2 cells. Under a laser confocal microscope (200x), the fluorescence intensity was detected with DAPI (blue), p-AKT antibody (green), and AKT antibody (red).

As shown in Figure 9, DAPI emits blue fluorescence upon binding to the nucleus, and the intensity of green fluorescence and red fluorescence represents the expression level of p-AKT and AKT, respectively. Compared with the normal group of cells, the expression of p-AKT was significantly inhibited in the OA-induced cells. While compared with the model group, the fluorescence intensity of p-AKT was strengthened with an increasing dose of PCE, indicating that the mechanism of PCE for preventing and treating hyperlipidemia might be related to the enhancement of AKT phosphorylation. The expression of AKT in OA-treated cells appeared to be decreased but not statistically significant. Subsequent WB experiments confirmed the above results, as shown in Figure 8(b). Compared with normal cells, after 24 hours of OA induction, the effect of AKT phosphorylation in HepG2 cells was significantly inhibited, while no significant changes in the expression of AKT were evident. However, compared with the model group, PCE (5, 10, and 20 *μ*g/mL) treatment significantly upregulated the function of AKT phosphorylation in HepG2 cells. Importantly, PCE also significantly favored the phosphorylation of PI3K, and no significant difference was noted in PI3K expression. These results suggested that PCE might activate the PI3K/AKT pathway to exert a protective effect on OA-stimulated HepG2 cells, showing an antihyperlipidemic effect.

**Figure 10:**
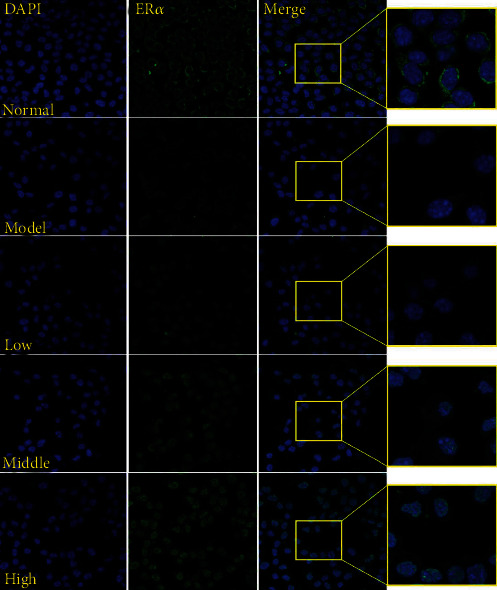
The effect of PCE on the expression of ER*α* in HepG2 cells. Under a laser confocal microscope (200x), the fluorescence intensity was detected with DAPI (blue) and ER*α* antibody (green).

In addition, as shown in Figure 8(b), compared with the normal group, the expression of ER*α* protein in the model group was significantly reduced; and compared with the model group, the expression of ER*α* protein in the cells gradually increased after treatment with different doses of PCE. As shown in Figure 10, the immunofluorescence experiments demonstrated the same results.

## 4. Discussion

Increasing evidences have suggested that extracts/monomers from herbal medicines are beneficial for the health of human being [15, 16]. Modern studies have shown that PCE can ameliorate blood glucose and lipid metabolism and exert significant effects on the treatment of metabolic syndrome. Multiple lines of evidence demonstrate that the stilbene (polydatin, resveratrol, etc.) in the active ingredients of PCE has a significant regulatory effect on lipid metabolism [17–19]. However, there is no systematic research on the pharmacological effects of PCE on improving hyperlipidemia. In this study, we have first conducted in vivo experiments using high-fat diet-induced hyperlipidemia rat models to verify whether PCE has an antihyperlipidemic effect. The results have shown that the serum levels of TC, TG, LDL-C, HDL-C, and ox-LDL in the hyperlipidemia group were higher than those in the normal control group, while PCE intervention could ameliorate the pathological state of hyperlipidemia rats and pathological changes in the liver of rats.

To further explore the active ingredients of PCE against hyperlipidemia and its mechanism of action, we have established a “drug-active ingredient-target-disease” network and analyzed related pathways. The results suggested that the antihyperlipidemic effects of PCE are achieved by regulating the expression of 27 genes by its 12 main active ingredients. At the same time, we also preliminarily explored the lipid-lowering effect of these potentially active compounds in PCE through in vitro cell model, which was consistent with our previous network pharmacological results. In other words, resveratrol and polydatin could significantly reduce lipid formation in OA-induced HepG2 cells. In addition, we have performed protein interaction network, GO function, and KEGG pathway enrichment analysis to decipher the 27 potential antihyperlipidemic targets of PCE, and the results demonstrate that 27 target proteins are mainly involved in the regulation of signaling pathways such as FOXO, PI3K, and estrogen, while targets such as FOXO3 and ES*α* are key targets in PPI. Taken together, our data suggest the active components of PCE may exert antihyperlipidemic effects via ameliorating lipid metabolism by participating in the regulation of the PI3K/AKT/FOXO signaling pathway.

OS can cause lipid peroxidation of cell membranes, produce propylene glycol and *β*-hydroxylated nonene, inhibit the conversion of TG to LDL-C, and suppress the fatty acid transport, leading to fat accumulation [20, 21]; MDA is a final product of lipid metabolism. As a result, the antioxidant capacity is impaired and the MDA concentration increases [22]. The free radicals produced can severely damage the structure of liver cell membranes and cause swelling and necrosis of liver cells. SOD, CAT, and GSH-Px are the primary antioxidant enzymes in endogenous antioxidants [23], SOD can catalyze O_2_-·dismutation to produce H_2_O_2_ and O_2_ at a rate significantly higher than that of physiological spontaneous decomposition [22], reduce and block lipid peroxidation, and exert a protective effect on liver cells. H_2_O_2_ is continued to decompose into H_2_O and O_2_ by CAT [23], protecting cells from H_2_O_2_ damage. GSH-Px is an enzyme that catalyzes the reduction of peroxides and hydroxyl radicals and requires glutathione as an auxiliary substrate, by oxidizing reduced GSH to glutathione disulfide and then reducing it to GSH by glutathione reductase [23]. These enzymes play key regulatory roles in maintaining redox homeostasis [24]. However, external stimuli can easily break this homeostasis and cause OS. OS is defined by an imbalance between increased levels of reactive oxygen species (ROS) and a low activity of antioxidant mechanisms [25]. Low concentration of ROS is essential for transmitting cell signals, while high concentration of ROS can cause damage to cellular macromolecules (such as DNA, lipids, and proteins), which will eventually lead to cell necrosis and apoptosis [26]. Therefore, paying attention to OS may be another way to prevent and treat hyperlipidemia. In the in vitro experiments, we have found that PCE can effectively reduce OA-induced adipogenesis in HepG2 cells, reduce the levels of TG and MDA in the hyperlipidemia cell model, increase GSH content and GSH-px, CAT, and SOD enzyme activity, and improve the level of cellular OS. In addition, we have confirmed that PCE can reduce OA-induced ROS production in HepG2 cells. These results indicate that polydatin can effectively correct the OS state in adipocytes induced by OA, reduce lipid peroxidation, and prevent fat accumulation.

The target genes of FOXO include manganese superoxide dismutase and catalase, which are involved in combating OS in multiple cell types. The markers of OS are elevated in adipose tissue, indicating that OS may be one of the causes of hyperlipidemia. However, OS is caused by the imbalance between the production of ROS and the production of antioxidant enzymes, so the production of FOXO indirectly affects the occurrence of hyperlipidemia. The human ortholog of SIR2 is SIRT1, which forms a complex with FOXO3a in response to OS. Interestingly, SIRT1 promotes the activation of FOXO3a to resist OS but induces cell cycle arrest and inhibits FOXO3a-induced cell death [27]. These results indicate that the SIRT1-FOXO3a pathway may play a protective role in fat cell dysfunction caused by obesity [28]. In addition, Zhao and others confirmed that polydatin can effectively reduce the blood lipid level of hyperlipidemia rats and improve liver fat lesions. The mechanism may be correlated with the reduction of fatty acid and cholesterol synthesis and the improvement of OS state in hyperlipidemia rats. Thereby, lipid peroxidation is decreased to prevent fat accumulation [29]. Ming et al. have reported that Cangju Qinggan Jiangzhi Recipe can improve liver damage induced by MCD diet in steatohepatitis by regulating the SIRT3-FOXO3 signaling pathway [30].

OS and inflammation are inseparable, inflammation can cause OS, and OS can also cause inflammation. Therefore, when talking about OS, the role of inflammation should not be ignored. In recent years, studies have shown that FOXO3*α* also plays an important inhibitory role in inflammation. The main reason may be by regulating the number and function of mononuclear macrophages or inhibiting the excessive activation of mononuclear macrophages, downregulating the NF-*κ*B pathway in the inflammatory response, and inhibiting the secretion of Th1 and Th2 inflammatory factors [31]. Previous studies have shown that the PI3K/AKT pathway regulates lipid metabolism and may cause excessive lipid deposition in liver cells when the function of FOXO3, a downstream molecule of PI3K/AKT, is blocked. FOXO3*α* is also a downstream molecule of the P13K/AKT signaling pathway [32]. When inflammatory signals activate P13K/AKT, it can induce AKT to combine with FOXO3*α* in the nucleus, and phosphorylated FOXO3*α* separates from the DNA binding site into the cytoplasm, thereby reducing its transcriptional activity and blocking the transduction of the TLR4 signaling pathway [33]. When pathogens activate the body's immune system, FOXO3*α* can inhibit the production of inflammatory cytokines such as TNF-*α* and IL-6. Therefore, FOXO3*α* plays an important biological role in regulating immune-mediated inflammation [31].

These studies also suggest that intervention in the PI3K/AKT/FOXO3 signaling pathway is of great significance for the treatment of hyperlipidemia. In our study, western blot and immunofluorescence experiments have verified that PCE has a significant regulatory effect on the PI3K/AKT/FOXO3 signaling pathway in OA-induced HepG2 cells.

In addition, estrogen can affect lipoprotein metabolism and inflammatory markers, which may be impaired by the changes in the expression and function of estrogen receptor *α* (ER*α*/ESR1) [34]. Moreover, estrogen can directly inhibit liver TG synthesis by activating Er*α* [35]. In this study, we have found that PCE can also activate ER*α* in the hyperlipidemia cell model to reduce blood lipids.

In summary, this study has revealed the potential active components of PCE against hyperlipidemia and their possible mechanism of action, providing a basis for further research on the effective material basis of PCE against hyperlipidemia and the development of antihyperlipidemia components of Chinese material medica.

## Figures and Tables

**Table 1:** The plan of drug intervention.

Group	Dosage	Mode of administration
Normal	Equal volume CMC-Na	i.g., 4 weeks
Model	Equal volume CMC-Na	i.g., 4 weeks
Positive	1.8 mg/kg fenofibrate	i.g., 4 weeks
L	90.0 mg/kg PCE	i.g., 4 weeks
M	180.0 mg/kg PCE	i.g., 4 weeks
H	360.0 mg/kg PCE	i.g., 4 weeks

**Table 2:** Precursor and product ions of constituents in *Polygonum cuspidatum Sieb.et Zucc*.

No.	Compound name	*t*_R_/min	Molecular formula	[M-H]^−^	[M+H]^+^	MS/MS *m*/*z*
1	Ellagic acid	3.61	C_14_H_6_O_8_	300.9995		257.0193, 228.0068, 185.0241
2	Polydatin	4.01	C_20_H_22_O_8_	389.1243		227.0859, 143.0497
3	Epicatechin gallate	4.21	C_22_H_18_O_10_	441.0836		
4	Resveratrol	10.81	C_14_H_12_O_3_	227.0712		142.9914, 185.0603
5	Cynaroside	11.27	C_21_H_20_O_11_	447.0942		285.0428, 256.0375, 212.0472, 108.3744
6	Glycitein	12.56	C_16_H_12_O_5_		285.0758	270.0519, 242.0573, 183.0803
7	Isokaempferide	15.45	C1_6_H_12_O_6_		301.0709	283.0602, 255.0653, 226.0621, 128.0622
8	Luteolin	17.71	C_15_H_10_O_6_	285.0454		257.0454, 242.0223, 213.0557, 109.8052
9	Genistein	19.15	C_15_H_10_O_5_	269.0458		241.0504, 225.0556
10	Formonontin	19.50	C_22_H_22_O_9_	267.0294		225.4558, 197.1059
11	Emodin	26.20	C_15_H_10_O_5_		271.0603	225.0544, 183.0809
12	Marmesin	26.69	C_14_H_14_O_4_		247.0968	229.0859

**Table 3:** Topological parameters of the compound-target network.

Name	Degree	Average shortest path length	Betweenness centrality	Closeness centrality
ESR1	11	1.775	0.105266	0.56338
C4	10	2.2	0.063913	0.454545
MAOA	8	1.925	0.050444	0.519481
C5	6	2.4	0.021826	0.416667
C7	6	2.4	0.021826	0.416667
C8	6	2.4	0.018167	0.416667
MGAM	5	2.075	0.022493	0.481928
PTK2	5	2.075	0.021141	0.481928
MMP1	5	2.075	0.02669	0.481928
C1	5	2.45	0.012483	0.408163
C9	5	2.45	0.012703	0.408163
C10	5	2.45	0.012703	0.408163
C11	5	2.45	0.01388	0.408163
C2	4	2.5	0.013482	0.4
C3	4	2.5	0.013482	0.4
C6	4	2.5	0.01237	0.4
C12	3	2.55	0.008768	0.392157
GNB5	2	2.225	0.0047	0.449438
PIK3R3	2	2.225	0.006274	0.449438

## Data Availability

The datasets generated for this study are available on request to the corresponding authors.
